# The genetics of primary dysmenorrhoea: a systematic review

**DOI:** 10.1530/RAF-26-0004

**Published:** 2026-07-30

**Authors:** Ella Heger, Miriam Szabo, Rachel Reid-McCann, Ioana-Wilhelmina Lucinescu, Nura Fitnat Topbas Selcuki, Krina T Zondervan, Katy Vincent

**Affiliations:** ^1^ St Anne’s College, University of Oxford, Oxford, UK; ^2^ Nuffield Department of Women’s and Reproductive Health, University of Oxford, Oxford, UK; ^3^ Istanbul Hamidiye Etfal Training and Research Hospital, University of Health Sciences Turkey, Istanbul, Turkey

**Keywords:** primary dysmenorrhoea, dysmenorrhoea, pain, menstruation, genetics, genetic predisposition to disease, genome-wide association study, systematic review

## Abstract

**Abstract:**

Dysmenorrhoea is the most common gynaecological condition in women of reproductive age. Approximately 90% of cases are primary dysmenorrhoea (PDM), which is not associated with an underlying pathology. Dysmenorrhoea can have a substantial impact on quality of life, yet current therapies do not provide adequate relief to everyone. Family history (FHx) is a known risk factor that suggests genetic involvement. We therefore aimed to synthesise the literature describing the genetics of PDM. Three databases (MEDLINE, Embase and Web of Science) were searched for studies published up to November 2025. Key search terms included ‘dysmenorrhea’, ‘polymorphism’ and ‘SNP’. Articles were screened independently by two researchers, and data were extracted and presented in a narrative format. Quality assessment was conducted using the Newcastle–Ottawa Scale and STREGA guidelines. Fifteen articles were included in the review (*n* = 7 case-control; *n* = 4 cross-sectional; and *n* = 4 genome-wide association studies (GWASs)). Candidate gene studies identified seven polymorphisms and two combined genotypes that were associated with PDM. One significant positive association was replicated in two studies (*ESR1 *PvuII). GWASs highlighted loci near the *NGF*, *ZMIZ1, IL1A *and* IL1B* genes. The *NGF* and *IL1* loci support what is known about the involvement of inflammatory pathways in PDM pathogenesis. However, they are also known endometriosis loci. The current literature is limited by a lack of adequate secondary dysmenorrhoea case exclusion, limited ethnic populations and low quality. Further research in multiple ancestries is essential to increase generalisability, while thorough diagnostic protocols will reduce misclassification, improve understanding of PDM pathogenesis and thus help inform new treatment development.

**Lay summary:**

Period pain is extremely common in menstruating women and has a major impact on their lives, yet we still do not have a full understanding of its biological causes or how to treat all women effectively. Research shows that period pain can run in families; therefore, we conducted this review to highlight which genes appear to be related to period pain (specifically period pain that is not caused by an existing condition, such as endometriosis or fibroids). After searching three academic databases, we found 15 studies that met our criteria. Three genes were highlighted across two or more studies; these genes are known to be involved in pain processing and how the body deals with immune responses and oestrogen (a female hormone). From this review, we know that we need more research on this topic as there are still likely to be more genes involved in period pain and these might vary in different ethnic populations.

## Introduction

Dysmenorrhoea (period pain) is the most common gynaecological condition in women of reproductive age (Iacovides *et al.* 2015). It is characterised by cyclical menstrual pain, which may be accompanied by headaches, nausea, vomiting, diarrhoea, bloating or fatigue (Iacovides *et al.* 2015). Prevalence rates for dysmenorrhoea range from 16 to 91% (Ju *et al.* 2014) or from 2 to 36% for severe pain (Söderman *et al.* 2019). It has a substantial impact on school or university attendance, social activities and quality of life (Söderman *et al.* 2019) as well as significant economic consequences (Ju *et al.* 2014). However, despite its high prevalence and personal and societal impacts, dysmenorrhoea is generally understudied, and the aetiology is not yet fully understood.

Risk factors for dysmenorrhoea include smoking, longer bleeding duration, heavy menstrual flow and high stress levels (Tavallaee *et al.* 2011, Muluneh *et al.* 2018). Family history (FHx) has also been recognised as an important risk factor (Ozerdogan *et al.* 2009, Iacovides *et al.* 2015, Muluneh *et al.* 2018). This is usually defined as having a first-degree relative (mother, sister) with dysmenorrhoea and can increase the risk of dysmenorrhoea 3.5-fold compared to individuals with no FHx (Ozerdogan *et al.* 2009). Women with FHx of dysmenorrhoea also tend to experience more severe pain (Tavallaee *et al.* 2011). This could be due to shared environments and learned behaviours regarding pain (Hu *et al.* 2020), but a genetic component is also likely, considering twin studies have previously estimated heritability to be 38% for menstrual pain (Silberg *et al.* 1987).

It is important to acknowledge that primary dysmenorrhoea (PDM) (no underlying pelvic pathology; comprising 90% of cases) (Gutman *et al.* 2022) is likely to have distinct risk factors and pathogenesis to secondary dysmenorrhoea (SDM) (pain associated with an underlying disease, for example, endometriosis, adenomyosis, fibroids or pelvic inflammatory disease) (Durand *et al.* 2021). While a laparoscopy is the only definitive method for confirming the diagnosis of PDM (by excluding SDM causes not visible on imaging), many studies report a PDM diagnosis on the basis of clinical judgement, e.g. using one or a combination of history taking, clinical examination or ultrasonography, potentially skewing the research focused on PDM.

Currently, pharmacological treatments for PDM are limited to hormonal therapies (usually aiming to suppress menstruation or prevent thickening of the uterine lining) or non-steroidal anti-inflammatory drugs (NSAIDs). NSAIDs have been shown to be 4.4 times more effective than placebo in treating PDM (Marjoribanks *et al.* 2015), supporting the theory that overproduction of prostaglandins, particularly PGF2a and PGE2, contributes to the pathogenesis of dysmenorrhoea (Iacovides *et al.* 2015). However, current treatment strategies do not provide adequate relief for all who experience dysmenorrhoea, and there is an increasing move away from hormone therapies, especially among adolescents and young adults (Hellström *et al.* 2019). Thus, novel therapies are urgently needed. A better understanding of the genetic determinants of dysmenorrhoea is key to informing the development of new therapies. Advancing knowledge in this area is likely to have a far-reaching impact given the influence of dysmenorrhoea on all domains of life and the increasing evidence that menstrual pain is associated with adverse long-term health outcomes, such as mental health diagnoses (Zhao *et al.* 2021) and chronic pain (Reid-Mccann *et al.* 2025).

This systematic review aims to summarise the current evidence for determining the associations between gene polymorphisms and PDM to further inform the field.

## Methods

This review was performed in accordance with the Preferred Reporting Items for Systematic Reviews and Meta-Analyses (PRISMA) statement (Page *et al.* 2021), and the protocol was registered on PROSPERO (registration ID: CRD42023455337) on 17 August 2023. 

### Literature search

A comprehensive search strategy was designed by EH, MS and KV with advice from a medical librarian, using key terms relating to dysmenorrhoea and genetic factors, for example, ‘dysmenorrhea’, ‘dysmenorrhoea’, ‘menstrual pain’, ‘polymorphisms’ and ‘SNPs’. The search strategy was subsequently trialled and iteratively optimised via multiple pilot searches, which were cross-checked against a list of target articles collated from a preliminary exploration of the literature. Final searches were conducted in October 2023 using three databases (MEDLINE (Ovid), Embase (Ovid) and Web of Science Core Collection). The search strategy used in MEDLINE can be seen in Supplementary Fig. 1 (see section on Supplementary materials given at the end of the article).

Auto-alerts were set up following the October 2023 search so that, as new articles were added to the databases, the searches were automatically re-run to identify any additional relevant articles. Any such articles were evaluated and screened until November 2025.

### Screening and eligibility criteria

Search results were retrieved and duplicates were removed, both automatically in Endnote 21 and manually by EH, when necessary. References were then imported into Rayyan (Ouzzani *et al.* 2016) for blinded screening. Two authors (EH and MS) independently screened the titles and abstracts (primary screening) and then read the full text of the remaining articles in the second screening stage. Each stage was followed by a conflict resolution meeting to resolve any differences in inclusion/exclusion of articles and establish a consensus. Additional articles identified up to November 2025 were screened by EH and then discussed with MS to validate their inclusion/exclusion.

All English language studies investigating specific genes/polymorphisms associated with PDM in females were included. No restriction was placed on year of publication. Non-human studies and studies not published in peer-reviewed journals were excluded. Full inclusion and exclusion criteria are listed in Table 1. Given that PDM is often poorly defined, with a lack of as yet unidentified pathology assumed to mean the lack of pathology present, we also included studies that did not differentiate between PDM and SDM. However, any articles relating only to SDM were excluded.

**Table 1:** Inclusion and exclusion criteria.

Include	Exclude
Humans – female only	Animal studies
PDM as defined by the study	SDM – e.g. confirmed diagnosis of endometriosis, adenomyosis or any other identifiable pelvic pathologies
○ If the study does not differentiate between PDM and SDM, it will still be included	
Age range: menarche to end of life	Dysmenorrhoea due to intrauterine contraceptive device
Study design of any type	Cancer diagnosis
Studies presenting original data regarding specific genes/polymorphisms involved in PDM in females	Studies not published in peer-reviewed journals – grey literature, conference abstracts, PhD theses, pre-prints, case reports, letters, editorials, reviews, opinion pieces
	Articles not in English language

PDM, primary dysmenorrhoea; SDM, secondary dysmenorrhoea.

### Data extraction and quality assessment

The relevant data from eligible studies were extracted following the second screening stage. The following data were extracted and tabulated: paper title; first author; year published; ethnic origin of participants; country of study; study design/type of study; how PDM is defined; inclusion criteria for PDM subjects; how SDM cases were excluded; how healthy controls were defined; number of participants; number of controls; mean age of participants; mean age of controls; method of genotyping; statistical analysis software used; genetic associations found (results of the study); other significant variables associated with primary dysmenorrhoea; and author conclusions.

The quality of the included studies was also evaluated at this stage to assess the risk of bias. The STREGA guidelines (Little *et al.* 2009) were used for every study, and the Newcastle–Ottawa Scale (NOS) (Wells *et al.* 2000), in addition, for case–control studies. The NOS involves three sections, in which stars are awarded for fulfilling certain criteria – selection of cases and controls (max. four stars); comparability of cases and controls (max. two stars); and ascertainment of exposure (max. three stars). Studies were ranked as having a high (0–3 stars), medium (4–6 stars) or low (7–9 stars) risk of bias. All studies were included in the review regardless of the methodological quality assessed by these tools.

Data extraction and quality assessment were carried out by one investigator (EH) and, upon completion, checked independently by MS.

### Data synthesis and analysis

A narrative synthesis was conducted, summarising the significant genetic variants (*P* < 0.05 for candidate gene studies; *P* < 5 × 10^−8^ for GWASs) identified as being associated with PDM, as well as their specific genetic location and known or potential biological function. Meta-analysis was planned for genetic variants, including subgroup analysis based on ethnicity, if sufficient data were available.

## Results

### Search and screening results

**Figure 1:**
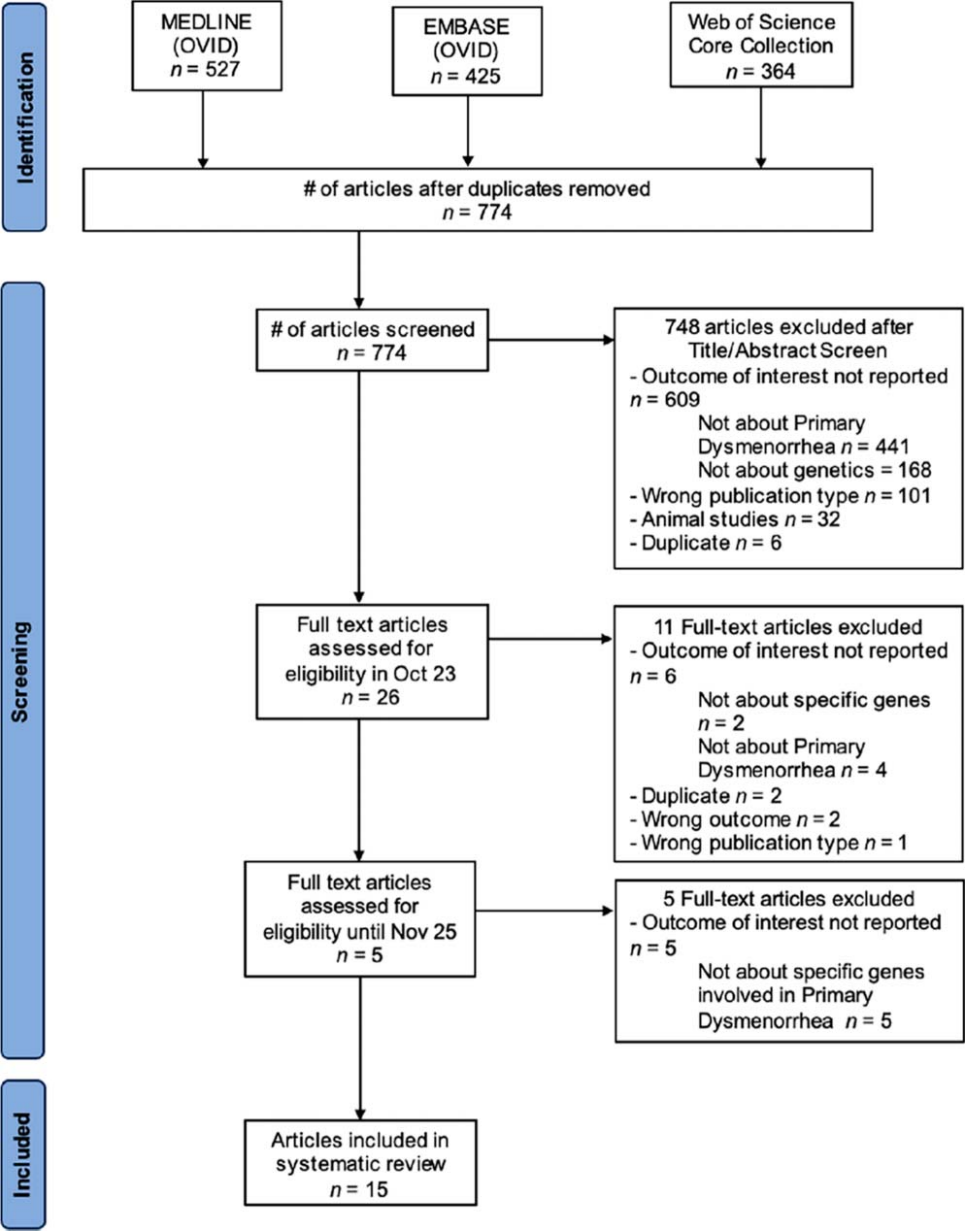
Flow chart detailing identification, screening and inclusion of studies in this review.

The original search (October 2023) yielded 774 articles after duplicates were removed. Publication year ranged from 1950 to 2023. A total of 748 articles (97%) were excluded during the title/abstract screening, meaning that 26 articles progressed to the second screening stage. After a full-text review, a further 11 articles (42%) were excluded, leaving 15 articles to be included in the systematic review. Evaluation of the search auto-alerts until November 2025 identified five potentially relevant articles (Nacar *et al.* 2023, Chen *et al.* 2024, Hsu *et al.* 2024, Li *et al.* 2024, Liu *et al.* 2024); however, none of these met the inclusion criteria following a full-text review. A detailed search and screening history is shown in Fig. 1.

Of the 15 included studies, seven (46.7%) were case–control studies, four (26.7%) were cross-sectional studies, and four (26.7%) were GWASs.

### Characteristics of case–control and cross-sectional (candidate gene) studies

The characteristics of the included case–control and cross-sectional studies are summarised in Table 2. Five studies involved Turkish participants (Ozsoy *et al.* 2015, Dogru *et al.* 2016, Ozsoy *et al.* 2016, Esen *et al.* 2020, Nacar *et al.* 2022), two studies had a Nigerian population (Olasore *et al.* 2022, Olasore *et al.* 2023), and there was one study with each of the following populations: Taiwanese (Lee *et al.* 2014), Chinese (Wu *et al.* 2000), Asian (Donayeva *et al.* 2023) and South Korean (Woo *et al.* 2010). It is important to note that Olasore *et al*. (2022) make reference to over 250 different ethnic groups within Nigeria, so their population is likely to be diverse. The mean age of participants ranged from 15 to 26 years. Two studies were conducted in adolescents (aged ≤ 17 years) (Woo *et al.* 2010, Donayeva *et al.* 2023). One study did not report the mean age of participants (Wu *et al.* 2000). For case–control studies, sample sizes ranged from 200 (Lee *et al.* 2014) to 302 (Ozsoy *et al.* 2016) (Table 3). For cross-sectional studies, sample sizes ranged from 102 (Olasore *et al.* 2022) to 435 individuals (Wu *et al.* 2000) (Table 3).

**Table 2:** Characteristics of case–control and cross-sectional (candidate gene) studies.

Reference	Region	Cases		Controls		Definition of PDM	Exclusion of SDM	Other exclusion criteria
		*n*	Age*	*n*	Age*			
Lee *et al.* (2014)	Taiwan	99	23.3	101	23.8	Pain (>4 on NRS) associated with menstruation in the absence of organic pelvic disease, lasting >6 months	Organic pelvic disease visible by ultrasound	Irregular, short or long menstrual cycle; OCP use within 6 months; previous childbirth; inconsistent pain intensity
Ozsoy *et al.* (2015)	Turkey^a^	159	25.5	135	NR	Recurrent painful menses within past 5 years that did not respond well to NSAIDs	Known gynaecological disorders or SDM diagnoses excluded	Irregular, short or long menstrual cycle; PCOS; pelvic surgery; GI, gynaecological or autoimmune disorders
Dogru *et al.* (2016)	Turkey^a^	154	25.5	144	26.0	Recurrent painful menses within past 5 years that did not respond well to NSAIDs	Diagnosis of endometriosis, uterine myoma or adeno-myoma; gynaecological abnormality or pelvic pathology identified by clinical examination or ultrasound	Irregular, short or long menstrual cycle; history of surgery; hormonal, radio- or chemotherapy; serious psychiatric or medical disorder
Ozsoy *et al.* (2016)	Turkey^a^	152	25.5	150	26.0	Recurrent painful menses within past 5 years	Diagnosis of endometriosis, uterine myoma or adeno-myoma; gynaecological abnormality on examination	Irregular, short or long menstrual cycle; history of pelvic surgery; GI, gynaecological or autoimmune disorders
Esen *et al.* (2020)	Turkey^b^	120	25.6	116	25.8	Recurrent painful menses within past 5 years	Gynaecological pathology on examination	Irregular, short or long menstrual cycle; history of pelvic surgery; GI, gynaecological or autoimmune disorders
Nacar *et al.* (2022)	Turkey^c^	122	25.6	150	25.9	Painful menses within past 5 years	Gynaecological pathology on examination	Irregular, short or long menstrual cycle; history of pelvic surgery; GI, gynaecological or autoimmune disorders
Donayeva *et al.* (2023)	Central Asia^d^	205	14.8	210	15.2	Painful menstruation (≥5 on VAS for ≥1 year since menarche)	Trans-abdominal pelvic ultrasound used to exclude pelvic pathology	Obesity; previous pelvic surgery; neurological or psychiatric disorders; hormonal therapy within the past year
Wu *et al.* (2000)	China	129	NR	306	NR	Pelvic or lower abdominal pain associated with menstruation	Physician-diagnosed organic diseases, such as endometriosis, adhesive disease or other uterine pathology	Smoking or consuming alcohol
Woo *et al.* (2010)	South Korea	172	^†^	30	^†^	Pelvic or lower abdominal pain associated with menstruation during every period	Suspected organic pelvic disease due to i) heavy and irregular menses; ii) no response to NSAIDs; iii) pain not associated with menstruation	Surgery, chemotherapy, radiation therapy, pregnancy, exogenous hormones or treatment for obstetric/gynaecological disease in past 3 months
Olasore *et al.* (2022)	Nigeria	67	^‡^	35	^‡^	NR	NR	NR
Olasore *et al.* (2023)	Nigeria	167	^⁋^	72	^⁋^	Pelvic, lower abdominal or lower back pain, which is associated with the menstrual period	Any history of gynaecological disease – e.g. endometriosis, fibromyoma, ovarian cyst	Previous pregnancy; previous major pelvic surgery; use of OCPs

* Values are mean age in years.

^†^ All participants were aged between 16 and 17 years.

^‡^ Mean age of both cohorts was 20.6 years.

^⁋^ Mean age of both cohorts was 21.0 years.

^a^ Inner Central Black Sea region.

^b^ Black Sea region.

^c^ Middle Black Sea region.

^d^ Kazakhstan Aktobe region.

GI, gastrointestinal; NRS, numerical rating scale; NSAIDs, non-steroidal anti-inflammatory drugs; OCP, oral contraceptive pill; PCOS, polycystic ovary syndrome; VAS, visual analogue scale; PDM, primary dysmenorrhoea; SDM, secondary dysmenorrhoea.

**Table 3:** Findings from case–control and cross-sectional (candidate gene) studies.

Study/polymorphism (rsID)	Hypothesis	Exposure/risk group	Reference group	OR (95%CI)	*P* value
Wu *et al.* (2000)					
*CYP2D6^†^*	Hormone metabolism; pain-modulating pathways	*CYP2D6* Aa/aa	*CYP2D6* AA	1.7 (0.9–3.1)*	NR
*GSTM1^†^*	Oestrogen metabolism	*GSTM1* absent	*GSTM1* present	1.8 (1.0–3.4)*	NR
*CYP2D6^†^ GSTM1^†^* combined		*CYP2D6* Aa/aa and *GSTM1* absent	*CYP2D6* AA and *GSTM1* present	3.1 (1.2–8.0)*	NR
		*CYP2D6* AA and *GSTM1* absent		2.3 (1.0–5.1)*	NR
		*CYP2D6* Aa/aa and *GSTM1* present		2.9 (1.0–8.5)*	NR
Woo *et al.* (2010)					
*ESR1 *PvuII (rs2234693)	Oestrogen receptor regulation	PP + Pp	Pp	3.4 (1.4–8.2)	**0.007**
		PP	Pp + pp	1.5 (0.5–4.4)	0.490
*GSTM1^†^*	Oxidative stress detoxification	Null	Present	0.6 (0.3–1.4)	0.226
*GSTT1^†^*	Oxidative stress detoxification	Null	Present	1.3 (0.6–2.9)	0.495
*GSTP1* (rs1695)	Modulation of oxidative/inflammatory pathways	Ile/Val	Ile/Ile	2.0 (0.7–5.2)	0.178
		Val/Val	Ile/Ile	0.4 (0.004–4.4)	0.469
Lee *et al.* (2014)					
*BDNF* Val66Met (rs6265)	Central pain modulation	Met/Met genotype	Met/Val or Val/Val genotypes	2.27 (1.21–4.27)	NR
Ozsoy *et al.* (2015)					
*MTHFR* C677T (rs1801133)	Altered vascular function; inflammatory signalling and pain sensitivity	CC + CT genotypes	TT genotype	2.0 (0.8–5.5)	0.126
		CC genotype	CT + TT genotypes	1.4 (0.9–2.2)	0.168
		C allele	T allele	1.4 (0.9–2.0)	0.079
*IL4* intron 3 VNTR (rs79071878)	Immune response and alteration of anti-inflammatory capacity	P2P2 + P1P2	P1P1	1.1 (0.2–6.2)	0.886
		P2P2	P1P2 + P1P1	0.8 (0.4–1.3)	0.357
		P2 allele	P1 allele	0.8 (0.5–1.3)	0.440
Dogru *et al.* (2016)					
*MIF* -173 G>C (rs755622)	Pro-inflammatory signalling; prostaglandin production	GG genotype	GC + CC genotypes	1.4 (0.9–2.3)	0.194
		GG and GC genotypes	CC genotype	1.1 (0.3–4.1)	0.909
		G allele	C allele	1.3 (0.9–2.0)	0.239
*TNF* -308 G>A (rs1800629)	Cytokine production; increased inflammatory signalling	GG genotype	GA + AA genotypes	0.4 (0.2–0.8)	**0.009**
		GG + GA genotypes	AA genotype	0.6 (0.1–2.4)	0.650
		G allele	A allele	0.5 (0.3–0.8)	**0.009**
Ozsoy *et al.* (2016)					
*ESR1* -351 A>G (XbaI) (rs9340799)	Oestrogen receptor regulation	AG and GG genotypes	AA genotype	0.93 (0.57–1.51)	0.769
		GG genotype	AA and AG genotypes	1.73 (0.94–3.23)	0.076
		G allele	A allele	1.13 (0.82–1.57)	0.445
*ESR1* -397 T>C (PvuII) (rs22346939)	Oestrogen receptor regulation	TC and CC genotypes	TT genotype	1.29 (0.77–2.16)	0.328
		CC genotype	CT and TT genotypes	2.60 (1.40–4.95)	**0.002**
		C allele	T allele	1.46 (1.06–2.01)	**0.021**
*IL6* -572 G>C (rs1800796)	Cytokine production; increased inflammatory signalling	GC and CC genotypes	GG genotype	0.63 (0.36–1.10)	0.140
		CC genotype	GC and GG genotypes	0.48 (0.14–1.63)	0.365
		C allele	G allele	0.62 (0.38–1.01)	0.074
*IL6* -597 G>A (rs1800797)	Cytokine production; increased inflammatory signalling	AA or AG genotypes	GG genotype	1.28 (0.79–2.07)	0.371
		AA genotype	AG or GG genotypes	1.51 (0.52–4.35)	0.617
		A allele	G allele	1.17 (0.78–1.76)	0.502
*ESR1* -351A>G/-397T>C combined	Oestrogen receptor regulation	AG–TC combined genotype	NR	NR	**0.027**
		AA–TT, AA–TC, AA–CC, AG–TT, GG–TT, GG–TC, GG–CC combined genotypes	NR	NR	>0.05
*IL6* -572G>C/-597G>A combined	Cytokine production; increased inflammatory signalling	GG–GG, GG–GA, GG–AA, GC–GG, GC–GA, GC–AA, CC–GG, CC–GA genotypes	NR	NR	>0.05
Esen *et al.* (2020)					
*IL4* intron 3 VNTR (rs79071878)	Immune response and alteration of anti-inflammatory capacity	P2 allele	P1 allele	0.71 (0.43–1.15)	>0.05
		P1/P1 + P1/P2 genotypes	P2/P2 genotype	0.70 (0.38–1.16)	>0.05
		P1/P1 genotype	P1/P2 or P2/P2 genotypes	0.48 (0.01–6.38)	>0.05
Nacar *et al.* (2022)					
*PER3* VNTR (rs57875989)	Circadian regulation of hormonal and pain pathways	4 repeats	5 repeats	0.9 (0.6–1.2)	0.21
		4/4 + 4/5	5/5	0.7 (0.4–1.1)	**0.05**
		4/4	4/5 + 5/5	1.2 (0.6–2.2)	0.31
Olasore *et al.* (2022)					
*COX2* -1195 G>A (rs689466)	Elevated prostaglandin levels	AA or AG genotypes	GG genotype	3.07 (1.12–8.39)	**0.025**
		GG or AG genotypes	AA genotype	0.80 (0.19–3.32)	0.762
Donayeva *et al.* (2023)					
*VDR* TaqI (rs731236)	Vitamin D-mediated inflammatory regulation	T/T genotype	T/t or t/t genotypes	15.9 (0.9–280.4)	0.05
		T/t genotype	TT or t/t genotypes	1,367.2 (83.7–223,342.5)	**<0.0001**
		t/t genotype	TT or T/t genotypes	106.2 (6.48–1,739.5)	**0.001**
Olasore *et al.* (2023)					
*eNOS* Glu298Asp (rs1799983)	Nitric oxide availability	TT or GT genotypes	GG genotype	3.3 (1.8–5.9)	**<0.01**
		GG or GT genotypes	TT genotype	0.4 (0.2–0.9)	**<0.03**

*P* values in bold indicate polymorphisms significantly associated with PDM (*P* < 0.05).

* Summary statistic for association with recurrent dysmenorrhoea (no association found for occasional dysmenorrhoea – data not presented here).

^†^ rs# was not reported in paper and could not be determined after Google search. PDM, primary dysmenorrhoea.

It is important to note that five of the seven case–control studies (71%) were conducted by the same research group and recruited patients from the same hospital in the Central Black Sea region of Turkey (Ozsoy *et al.* 2015, Dogru *et al.* 2016, Ozsoy *et al.* 2016, Esen *et al.* 2020, Nacar *et al.* 2022). Therefore, it is possible that the same or a very similar population was used for each, especially given the almost identical mean ages of cases and controls (Table 2).

We assessed whether and how these studies differentiated between PDM and SDM in their populations. Among these 11 studies, nine differentiated between PDM and SDM (Wu *et al.* 2000, Lee *et al.* 2014, Ozsoy *et al.* 2015, 2016, Dogru *et al.* 2016, Esen *et al.* 2020, Nacar *et al.* 2022, Donayeva *et al.* 2023, Olasore *et al.* 2023). Three used ultrasonography (Lee *et al.* 2014, Dogru *et al.* 2016, Donayeva *et al.* 2023), while three others reported using a clinical examination only (Ozsoy *et al.* 2015, Esen *et al.* 2020, Nacar *et al.* 2022). The remaining three studies used history alone to exclude women with an existing diagnosis of conditions related to SDM, such as endometriosis, PCOS, myoma or ovarian cyst (Wu *et al.* 2000, Ozsoy *et al.* 2016, Olasore *et al.* 2023). No studies reported using laparoscopy to exclude SDM. Two studies made no attempt to differentiate between PDM and SDM (Woo *et al.* 2010, Olasore *et al.* 2022); therefore, it is likely that some SDM cases are present in these study cohorts.

### Findings from candidate gene studies

The results of the 11 case–control and cross-sectional studies are detailed in Table 3.

Twenty different polymorphisms were investigated across the 11 candidate gene studies, selected specifically by studies due to their known or suspected roles in oestrogen metabolism, cytokine, prostaglandin or other inflammatory signalling, circadian regulation of hormone and pain pathways, and pain modulation (Table 3). Three polymorphisms (*ESR1 *PvuII, *IL4* VNTR and *GSTM1*) were each investigated in two separate studies, but otherwise each study investigated a different polymorphism.

Two studies found that the *ESR1* -397T>C (PvuII) polymorphism was significantly (*P* < 0.05) associated with dysmenorrhoea. One study found that the C allele (OR: 1.46, 95% CI: 1.06–2.01, *P* = 0.021) and CC genotype (OR: 2.60, 95% CI: 1.40–4.95, *P* = 0.002) of the *ESR1* -397T>C (PvuII) polymorphisms were associated with an increased risk of PDM (Ozsoy *et al.* 2016). Woo *et al*. (2010) also reported that this polymorphism was associated with PDM under the dominant model (CC + CT:TT) (OR: 3.38, 95% CI: 1.39–8.21, *P* = 0.007), suggesting that the C allele in the *ESR1* polymorphism is associated with dysmenorrhoea.

Two studies found that the *IL4* intron 3 VNTR polymorphism was not associated with PDM. This was consistent in Ozsoy *et al.* (2015) using the P2P2 + P1P2:P1P1 genotype (OR: 1.1, 95% CI: 0.2–6.2, *P* = 0.886), P2P2: P1P2 + P1P1 genotype (OR: 0.8, 95% CI: 0.4–1.3, *P* = 0.357) or allele frequencies (OR: 0.8, 95% CI: 0.5–1.3, *P* = 0.440) (Ozsoy *et al.* 2015, Esen *et al.* 2020, also using the P1P1 + P1P2:P2P2 genotypes (OR: 0.67, 95% CI: 0.38–1.16, *P* > 0.05), P1P1:P1P2 + P2P2 genotypes (OR: 0.48, 95% CI: 0.01–6.38, *P* > 0.05) or allele frequencies (OR: 0.71, 95% CI: 0.43–1.15, *P* > 0.05) (Esen *et al.* 2020). The replication of this finding increases confidence in a true lack of effect; although the estimates are imprecise, sample sizes are small (*n* = 236–294), and generalisability is limited by both studies being conducted in a Turkish population.

With regard to the *GSTM1* polymorphism (either the presence or absence of *GSTM1*), two studies produced contrasting results. *GSTM1* absence was associated with recurrent PDM in one study (Wu *et al.* 2000) (adjusted OR: 1.8, CI: 1.0–3.4) (*n* = 435), and in the same study, *GSTM1* absence also increased the risk of recurrent PDM when combined with the *CYP2D6* Aa/aa (adjusted OR: 3.1, 95% CI: 1.2–8.0) or *CYP2D6* AA genotype (adjusted OR: 2.3, 95% CI: 1.0–5.1) (Wu *et al.* 2000). However, Woo *et al.* (2010) (*n* = 202) found that the *GSTM1*-absent polymorphism was not associated with dysmenorrhoea (unadjusted OR: 0.6, 95% CI: 0.3–1.4, *P* = 0.2) (Woo *et al.* 2010).

For the 17 polymorphisms investigated by a single study, six were found to be significantly (*P* < 0.05) associated with PDM: *BDNF* Val66Met (Lee *et al.* 2014), *TNF* -308G>A (Dogru *et al.* 2016), *PER3* VNTR (Nacar *et al.* 2022), *COX2* -1195G>A (Olasore *et al.* 2022), *VDR* TaqI (Donayeva *et al.* 2023) and *eNOS* Glu298Asp (Olasore *et al.* 2023). Two combined genotypes were also associated with PDM: *CYP2D6/GSTM1* variant genotypes (Wu *et al.* 2000) and *ESR1* AG-TC (heterozygous at XbaI and PvuII) (Ozsoy *et al.* 2016). In addition, eight of the investigated polymorphisms and one combined genotype were not found to be associated with dysmenorrhoea: *CYP2D6* (Wu *et al.* 2000); *GSTT1* (Woo *et al.* 2010); *GSTP1* (Woo *et al.* 2010); *MTHFR* C677T (Ozsoy *et al.* 2015); *MIF* -173G>C (Dogru *et al.* 2016); *ESR1* -351A>G (XbaI), *IL6* -572G>C and *IL6* -597G>A (Ozsoy *et al.* 2016); and the combined *IL6* -572G>C/-597G>A genotype (Ozsoy *et al.* 2016).

### Characteristics of GWASs

The characteristics of GWASs are summarised in Table 4. Participants in these studies were of European (Jones *et al.* 2016), Mainland Eastern Chinese (Li *et al.* 2017), Japanese (Hirata *et al.* 2018) and Taiwanese Han Chinese (Lee *et al.* 2022) ancestries. Total cohort sizes ranged from 6,770 (Li *et al.* 2017) to 15,206 (Lee *et al.* 2022), while the mean ages of cases and controls ranged from 19.9 and 19.4 years, respectively (Li *et al.* 2017), to 38.4 and 40.9 years, respectively (Lee *et al.* 2022). Two studies did not report mean age at group level for cases and controls (Jones *et al.* 2016, Hirata *et al.* 2018); however, Jones *et al.* (2016) reported that participants were between 18 and 45 years, while Hirata *et al.* (2018) had mean ages (SD) ranging from 31 (6.4) to 35 (7.0) when presented by pain severity level.

**Table 4:** Characteristics of genome-wide association studies (GWAS).

Reference	Study design	Ancestry	Number of			Mean age (years)		Definition of PDM	Exclusion of SDM	Other exclusion criteria
			Total	Cases	Controls	Cases	Controls			
Jones *et al.* (2016)	GWAS	European	11,891	10,106*	1,785*	^†^ ^,^ ^‡^	^†^ ^,^ ^‡^	No differentiation between PDM or SDM	None	Age > 45
								Dysmenorrhea was defined as average menstrual pain level before first child (4-point scale)		
Li *et al.* (2017)	2-stage GWAS and replication study	Mainland Eastern Chinese	6,770	3,082	3,688	19.9	19.4	Menstrual pain > 4 on VAS	Possible causes of SDM diagnosed or seen upon physical examination – e.g. endometriosis or other gynaecological problems	^†^
Hirata *et al.* (2018)	Meta analysis of 2 GWAS	Japanese	11,379	10,871^§^	477^§^	^¶^	^¶^	No differentiation between PDM or SDM	^†^	^†^
								Dysmenorrhea was defined as menstrual pain level before first child (5-point scale)		
Lee *et al.* (2022)	GWAS	Han Chinese in Taiwan	15,206	1,186	24,020	38.4	40.9	Frequent menstrual pain in the past 3 months or those with a clinical diagnosis of severe dysmenorrhea	Self-reported endometriosis, myoma uteri or PCOS	Age > 50; mental health disorder; occasional dysmenorrhea

* Study measured dysmenorrhea severity with an ordinal variable: 1,785 women reported no pain; 10,106 women reported at least some pain.

^†^ Not reported in study.

^‡^ Study reported that participants were between 18 and 45 years old.

^§^ Study measured dysmenorrhea severity using an ordinal scale: 477 women reported no pain; 10,871 women reported at least some pain.

^¶^ Age was presented for five dysmenorrhea severity levels: mean (SD) for no pain was 34.7(7.0) years; for the highest pain level, mean (SD) was 31.3 (6.4) years.

PCOS, polycystic ovary syndrome; VAS, visual analogue scale; PDM, primary dysmenorrhea; SDM, secondary dysmenorrhea.

In terms of differentiation between PDM and SDM cases, one study used clinical examination only (Li *et al.* 2017), while another excluded women with existing diagnoses of conditions related to SDM from their medical history (Lee *et al.* 2022). Two studies did not differentiate between PDM and SDM (Jones *et al.* 2016, Hirata *et al.* 2018).

### Findings from GWASs

#### Genetic loci associated with PDM identified in GWASs

Seven index SNPs reached genome-wide significance (GWS) (*P* < 5 × 10^−8^) in their respective studies (Table 5). All seven SNPs co-localised with three genes (*NGF*, *IL1* and *ZMIZ1*), with associations at *NGF* and *IL1* loci being replicated in at least one other study. All four GWASs found an association between SNPs located near the *NGF* locus and PDM – this represents the most widely validated association based on currently available data in this field. Notably, the associations with *NGF* were replicated across different ethnic groups (*n* = 3 East Asian and *n* = 1 European), while the findings relating to *IL1* were replicated in two East Asian populations (Hirata *et al.* 2018, Lee *et al.* 2022). Hirata *et al.* (2018) found that the two *NGF* SNPs that Jones *et al.* (2016) and Li *et al.* (2017) had identified in their studies (rs7523086 and rs752381, respectively) were in high linkage disequilibrium (LD, *r*^2^ > 0.8) with their GWS SNP (rs12030576). Similarly, Lee *et al.* (2022) reported that all three previously identified *NGF* SNPs were in high LD (*r*^2^ > 0.8) with the GWS SNP they found at this locus (rs2982742).

**Table 5:** Significant loci for primary dysmenorrhea from genome-wide association studies (GWAS).

Reference	Ancestry	Chromosome: signal range (*r*^2^ < 0.5)	Index SNP	Candidate gene(s)	Minor/effect allele (frequency in cohort)	OR (95% CI)	*P* value
Jones *et al.* (2016)	European	1*	rs7523086	*NGF*	A (0.33)	0.1 (0.07–0.13)^†^	4.1 × 10^−14^
Li *et al.* (2017)	Eastern Han Chinese	1*	rs76518691	*ZMIZ1*	A (0.13)	0.73 (0.66–0.81)	1.47 × 10^−9^
		10*	rs752831	*NGF*	C (0.54)	0.82 (0.77–0.88)	1.36 × 10^−8^
Hirata *et al.* (2018)	Japanese	1: 15.81–115.83 Mb	rs12030576	*NGF, RP4-663N10.1*	G*	1.52 (1.39–1.67)	1.13 × 10^−19^
		2: 113.48–113.58 Mb	rs80111889	*IL1, IL36RN, IL36B, IL37*	T*	1.53 (1.38–1.69)	1.90 × 10^−16^
Lee *et al.* (2022)	Han Chinese in Taiwan	1:115.80–115.83 Mb	rs2982742	*NGF*	T (0.51)	1.36 (1.25–1.48)	3.83 × 10^−13^
		2:113.55–113.58 Mb	rs11676014	*IL1A, CKAP2L, NT5DC4*	G (0.35)	1.32 (1.22–1.44)	7.43 × 10^−10^

* Not reported in study.

^†^ Value is effect size (95% CI) (per unit increase in dysmenorrhea severity on a 4-point ordinal scale).

Associations at *IL1* were reported by both Hirata *et al.* (2018) and Lee *et al.* (2022) – SNPs rs80111889 and rs11676014, respectively – with LD analysis by Lee *et al.* (2022) replicating Hirata *et al.*’s (2018) SNP finding. An association between *IL1A* gene (SNP rs3783550) and PDM was acknowledged by Li *et al.* (2017), but it did not reach GWS, after neither principal component analysis nor gene-based and pathway-based analyses (*P* = 2.6 × 10^−5^). Hirata *et al.* (2018), nevertheless, found this SNP (rs3783550) to be in LD with their *IL1* variant, suggesting some involvement in dysmenorrhoea.

The finding at *ZMIZ1* was not replicated, being identified only by Li *et al.* (2017). However, it is worth mentioning that all SNPs reported by Li *et al.* (2017) only reached significance after replication analysis in an independent cohort.

#### Functional analysis findings

Each GWAS also conducted a functional analysis of its GWS findings to identify the putative biological roles of GWS.

#### Variants located at the nerve growth factor (NGF) and RP4-663N10.1 genes

All GWASs identified index SNPs that were co-localised with the *NGF* gene, each of which were found to be non-coding (three intergenic (Jones *et al.* 2016, Li *et al.* 2017, Hirata *et al.* 2018) and one intronic to *NGF* (Lee *et al.* 2022)). Each study suggested putative regulatory functions of the identified genetic loci. Li *et al.* (2017) predicted that most SNPs (including the index SNP and those in LD) resided within promoter and/or enhancer elements, while Jones *et al.* (2016) also reported that half of SNPs in the cluster overlapped with promoter/enhancer histone marks in gynaecological tissues, suggesting that these variants lie within a regulatory domain that may control the gene expression. Lee *et al.* (2022) also predicted 21 of 24 SNPs within the *NGF*-situated cluster to alter regulatory binding motifs.

Jones *et al.* (2016), Hirata *et al.* (2018), and Lee *et al.* (2022) also reported the relevance of their GWAS hits to *RP4-663N10.1* – a conserved antisense long non-coding RNA (lncRNA) that spans the length of the *NGF* gene. In tissue expression analysis, Jones *et al.* (2016) reported highest expression of *RP4-663N10.1* in adipose tissue and gynaecological tissues, including the cervix, the fallopian tubes and the uterus. Similarly, Hirata *et al.* (2018) found moderate-to-strong support for the co-localisation of the index and LD SNPs with *RP4-663N10.1* eQTLs (expression quantitative trait loci) in adipose, ovarian and uterus tissues. eQTLs are genetic loci that regulate expression levels of mRNAs or proteins and thus explain some variation within a gene expression phenotype (Nica & Dermitzakis 2013). However, these studies found conflicting directions of effect, with Hirata *et al.* (2018) finding an increased expression of *RP4-663N10.1* in gynaecological tissues associated with increased dysmenorrhoea, while Jones *et al.* (2016) finding an increased dysmenorrhoea pain severity associated with reduced *RP4-663N10.1* expression. Fifteen SNPs in Lee *et al.* (2022) were eQTLs for *RP4-663N10.1* but in aortic tissue only.

#### Variants located at the interleukin-1 (IL1) gene

Hirata *et al.* (2018) found that *IL1* SNPs (rs80111889 and 42 high LD variants) were intronic to *IL1A* or intergenic regions between *IL1A* and *IL1B* or *IL1A* and *CKAP2L*. Strong support was found for co-localisation with *IL1A* eQTLs. Fourteen of the 42 high LD SNPs overlapped promoter/enhancer regions for ≥20 tissues. Lee *et al.* (2022) found that SNPs at the *IL1* locus were mostly located in intergenic regions and, similarly to Hirata *et al.* (2018), were predicted to have a regulatory function. A novel finding was that two SNPs at the *IL1B* locus were found to be eQTLs for *NT5DC4*, a gene within the NT5DC family that encodes a 5′-nucleotidase that catalyses hydrolysis of intracellular 5′ nucleotides (Singgih *et al.* 2021). Any relevance to dysmenorrhoea has not yet been explored (Cros-Perrial & Jordheim 2025).

#### Variants located at the zinc finger MIZ-type controlling 1 (ZMIZ1) gene

Tissue-specific regulatory analysis for the *ZMIZ1* index SNP (rs76518691) and its LD SNPs highlighted activity marks in adipose-derived mesenchymal stem cells (Li *et al.* 2017).

### Risk of bias (study quality)

All 15 studies were evaluated using the STREGA guidelines. Several checklist items were absent from many, if not all, studies, demonstrating that improvement in the reporting of genetics studies is needed. Surprisingly, the majority of studies did not report participant recruitment periods, with some also not specifying where the population was recruited from. Two further key areas lacking in reporting quality were the laboratory genotyping methods, where many failed to report where genotyping was done and how the DNA was stored prior to analysis, and any discussion of confounders or sources of potential bias by the authors. Some studies also failed to report genetic variants with widely used nomenclature, often lacking the rs#. Supplementary Table 1 provides a comprehensive overview of our STREGA evaluation of all 15 studies.

Of the seven case–control studies assessed using the NOS, five had high risk of bias and two had medium risk of bias. No studies adequately matched cases and controls or reported the non-response rate. Other common issues were studies not reporting where controls were recruited from and how PDM cases and controls were defined. The full NOS risk-of-bias assessment can be seen in Supplementary Table 2.

### Meta-analysis

There were insufficient data to undertake a meta-analysis as had been planned.

## Discussion

This review qualitatively summarises the current state of our understanding of the genetic associations with PDM. We identified evidence for loci at genes involved in inflammatory (*IL1*, *TNF*), pain (*NGF*, *BDNF*) and oestrogen metabolism (*ESR1*) pathways. Our review also highlights how poorly PDM is defined within studies and the limited data covering populations of mixed ethnicity, with only one transethnic finding (*NGF*). Since several of the gene findings have also been identified as endometriosis loci, this highlights how important proper differentiation between PDM and SDM is in order to understand their disease profiles and inform future treatments but also raises the important question of how distinct PDM and SDM really are.

Given that dysmenorrhoea is, by definition, a pain condition, it is perhaps unsurprising that the best replicated finding across studies was an association between *NGF* SNPs and PDM. NGF is a neurotrophin that stimulates nerve growth and has a well-established role in pain pathophysiology (Barker *et al.* 2020). Both pre-clinical and human studies have demonstrated a role for NGF in nociceptor sensitisation in both the short term and longer term. Importantly, in the context of dysmenorrhoea (which is related to contractions of the uterine muscle), NGF administration has been shown to be associated with the development of muscle pain (Lewin *et al.* 1993, Petty *et al.* 1994). In addition, NGF can trigger the release of inflammatory mediators and BDNF (Barker *et al.* 2020).

A recent large-scale GWAS for endometriosis (Rahmioglu *et al.* 2023) also identified a locus at the *NGF* gene. There is also evidence that NGF-containing peritoneal fluid from endometriosis patients increases neurite outgrowth (Barcena De Arellano *et al.* 2011). However, other studies are inconsistent in their findings relating NGF levels to endometriosis pain symptoms (Barcena De Arellano *et al.* 2013, Kajitani *et al.* 2013). BDNF has also been shown to relate to symptom severity and is suggested to have an important role in endometriosis-associated pain (Ding *et al.* 2018).

An established role of NGF in PDM could open the door to novel therapeutic opportunities, such as anti-NGF therapies. These have already been explored in other chronic pain conditions, including in women with interstitial cystitis/bladder pain syndrome (Nickel *et al.* 2016), a visceral pain phenotype with many features in common with dysmenorrhoea. However, significant adverse events, including abnormal peripheral sensation in trials of these therapies in osteoarthritis (Zhao *et al.* 2022), have led to the recommendation that more research and long-term follow-up are needed to address the current inconsistent, inconclusive safety outcomes.

The *ESR1 *PvuII polymorphism was also identified as a risk factor in two studies (Woo *et al.* 2010, Ozsoy *et al.* 2016); *ESR1* is known to be involved in oestrogen signalling. While there is a current lack of research on their role in PDM, *ESR1* in particular has been investigated in relation to endometriosis. Studies have demonstrated higher oestrogen receptor expression in endometriosis lesions of women with moderate-to-severe dysmenorrhoea, compared to those with absent or mild pain (Pluchino *et al.* 2020), potentially demonstrating a pain-specific role of oestrogen in dysmenorrhoea. Given that hormonal anti-oestrogen contraceptives are also used as treatment for PDM, the role of *ESR1* should be explored further. Interestingly, however, none of the PDM-focused GWASs identified *ESR1* as a significant variant, while endometriosis-focused GWASs have (Rahmioglu *et al.* 2023). This could be explained by the fact that studies included in this review likely failed to exclude all secondary dysmenorrhoea cases.

Inflammatory processes, particularly prostaglandins, have long been considered to be the key pathways involved in dysmenorrhoea, particularly PDM (Iacovides *et al.* 2015). Studies examining the inflammatory profiles of the menstrual effluent have demonstrated higher levels of both PGE2 (though not a consistent finding) and PGF2α in those with dysmenorrhoea compared to those without, in addition to other inflammatory mediators, including leukotrienes and other epoxy eicosatetraenoic acids (Powell *et al.* 1985, Bieglmayer *et al.* 1995). Interestingly, adolescents with dysmenorrhoea using NSAIDs had higher levels of PGF2α than those who did not use this medication, and this finding needs a more detailed investigation to better inform treatment paradigms (Kyathanahalli *et al.* 2025). Both IL1 and TNF cytokines stimulate prostaglandin production (Hertelendy *et al.* 2001, Barcikowska *et al.* 2020), as well as other inflammatory mediators and nitric oxide (Bradley 2008, Gabay *et al.* 2010). Therefore, a genetic association is biologically plausible. However, inflammation is also a key feature of SDM-related conditions, such as endometriosis and adenomyosis.

It is important to note that GWASs identify common genetic variants that do not have high penetrance in the population, meaning that the identified SNPs tend to have small effect sizes. For example, Jones *et al.* (2016) reported that the likelihood of PDM increased by 0.1 for each level of pain severity on a four-point scale for their *NGF* SNP. PDM is likely to be polygenic, and much more research is required before targeted interventions can be developed.

### Limitations

While this review was comprehensively conducted, several limitations remain. No grey literature and only English language studies were included, which may mean that some literature was missed. Sample sizes were relatively small in candidate gene studies (ranging from 102 to 435 participants), which may have limited the power needed to detect a significant association. Furthermore, five Turkish studies may have been conducted in the same clinical cohort; therefore, arguably, findings should have been subjected to a Bonferroni correction. Few GWASs have been conducted, and it is possible that these also required greater statistical power to detect associations. Studies were generally of low to medium quality. In addition, few ethnic groups were represented.

Importantly, there was evident inconsistency in the definition of PDM cases. Without clear exclusion of secondary causes, it is impossible to firmly assert that findings are associated with PDM rather than the presence of underlying pathology. As non-invasive diagnostic procedures for endometriosis improve, this challenge could be reduced for new cohorts, in which previous findings can then be replicated.

## Conclusion

This systematic review has evaluated the current literature on genetics and PDM, showing that both inflammatory and pain pathways appear to be involved. However, the literature is sparse and hampered by poor definitions of PDM and a lack of broad geographic and ethnic coverage, meaning that much remains unknown. Given the limited replicated results in this review, it is clear that further research in this field is urgently required. A better understanding of the pathogenesis of PDM will aid in the development of novel therapies and in identifying at-risk individuals, thus potentially allowing implementation of preventative strategies with the ultimate aim of reducing the burden of this common yet long neglected condition.

## Supplementary materials





## Declaration of interest

EH, IWL, MS, NFST and RRM have no competing interests to declare. KV received research grants from NIHR, NIH and EU IMI-2; received consultancy fees from Gedeon Richter, Bayer Healthcare, Reckitts and Gesynta; and was president of the IASP Special Interest Group on Abdominal and Pelvic Pain (until August 2024). KZ received research grants from NIHR, NIH, Gates Foundation, DoD US, EU Horizon, Aspira Labs Inc., Bayer AG, Chemo Research S.L., Proteomics International Pty Ltd and Roche Diagnostics GmbH and received a personal payment from Bayer AG until June 2022 as royalties associated with scientific collaboration between University of Oxford and Bayer. KZ was also an unpaid board member of the World Endometriosis Society until 2023 and is currently an unpaid board member of the World Endometriosis Research Foundation.

## Funding

This project did not receive external funding. RRM is currently funded by a grant from the Medical Research Foundation as part of the UKRI Strategic Priorities Fund (SPF) Advanced Pain Discovery Platform (APDP), a co-funded initiative by UKRI (MRC, BBSRC, ESRC), Versus Arthritis, the Medical Research Foundation and Eli Lilly and Company Ltd (grant ref: MR/W02697/X1). IWL received a postgraduate studentship from MRC-iCASE and Eli Lilly.

## Author contribution statement

KV and MS were involved in conceptualisation and methodology. EH and MS were involved in screening, data extraction and quality assessment. IWL, MS and RRM carried out supervision. EH wrote the original draft of the manuscript. All authors reviewed and edited the manuscript.

## References

[bib1] Barcena De Arellano ML, Arnold J, Vercellino F, et al. 2011 Overexpression of nerve growth factor in peritoneal fluid from women with endometriosis may promote neurite outgrowth in endometriotic lesions. Fertil Steril 95 1123–1126. (10.1016/j.fertnstert.2010.10.023) 21047631

[bib2] Barcena De Arellano ML, Arnold J, Lang H, et al. 2013 Evidence of neurotrophic events due to peritoneal endometriotic lesions. Cytokine 62 253–261. (10.1016/j.cyto.2013.03.003) 23545214

[bib3] Barcikowska Z, Rajkowska-Labon E, Grzybowska ME, et al. 2020 Inflammatory markers in dysmenorrhea and therapeutic options. Int J Environ Res Public Health 17 1191. (10.3390/ijerph17041191) 32069859 PMC7068519

[bib4] Barker PA, Mantyh P, Arendt-Nielsen L, et al. 2020 Nerve growth factor signaling and its contribution to pain. J Pain Res 13 1223–1241. (10.2147/jpr.s247472) 32547184 PMC7266393

[bib5] Bieglmayer C, Hofer G, Kainz C, et al. 1995 Concentrations of various arachidonic acid metabolites in menstrual fluid are associated with menstrual pain and are influenced by hormonal contraceptives. Gynecol Endocrinol 9 307–312. (10.3109/09513599509160464) 8629459

[bib6] Bradley JR 2008 TNF-mediated inflammatory disease. J Pathol 214 149–160. (10.1002/path.2287) 18161752

[bib7] Chen CY, Cheng CF, Chen PC, et al. 2024 Familial coaggregation and shared genetic influence between major depressive disorder and gynecological diseases. Eur J Epidemiol 39 1161–1170. (10.1007/s10654-024-01166-w) 39495462

[bib8] Cros-Perrial E & Jordheim LP 2025 A phenotypic journey into NT5DC proteins in cancer and other diseases. Exp Cell Res 446 114468. (10.1016/j.yexcr.2025.114468) 39971176

[bib9] Ding S, Zhu T, Tian Y, et al. 2018 Role of brain-derived neurotrophic factor in endometriosis pain. Reprod Sci 25 1045–1057. (10.1177/1933719117732161) 28954602

[bib10] Dogru HY, Ozsoy AZ, Karakus N, et al. 2016 Association of genetic polymorphisms in TNF and MIF gene with the risk of primary dysmenorrhea. Biochem Genet 54 457–466. (10.1007/s10528-016-9732-2) 27105877

[bib11] Donayeva A, Amanzholkyzy A, Nurgaliyeva R, et al. 2023 Vitamin D and vitamin D receptor polymorphism in Asian adolescents with primary dysmenorrhea. BMC Womens Health 23 414. (10.1186/s12905-023-02569-9) 37543584 PMC10403873

[bib12] Durand H, Monahan K & Mcguire BE 2021 Prevalence and impact of dysmenorrhea among university students in Ireland. Pain Med 22 2835–2845. (10.1093/pm/pnab122) 33822197 PMC8666000

[bib13] Esen M, Nursal AF, Duman E, et al. 2020 The role of Interleukin-4 VNTR polymorphism in dysmenorrhea development. Haseki Tıp Bülteni 58 364–369. (10.4274/haseki.galenos.2020.6162)

[bib14] Gabay C, Lamacchia C & Palmer G 2010 IL-1 pathways in inflammation and human diseases. Nat Rev Rheumatol 6 232–241. (10.1038/nrrheum.2010.4) 20177398

[bib15] Gutman G, Nunez AT & Fisher M 2022 Dysmenorrhea in adolescents. Curr Probl Pediatr Adolesc Health Care 52 101186. (10.1016/j.cppeds.2022.101186) 35523674

[bib16] Hellström A, Gemzell Danielsson K & Kopp Kallner H 2019 Trends in use and attitudes towards contraception in Sweden: results of a nationwide survey. Eur J Contracept Reprod Health Care 24 154–160. (10.1080/13625187.2019.1581163) 30920325

[bib17] Hertelendy F, Rastogi P, Molnár M, et al. 2001 Interleukin-1beta-induced prostaglandin E2 production in human myometrial cells: role of a pertussis toxin-sensitive component. Am J Reprod Immunol 45 142–147. (10.1111/j.8755-8920.2001.450304.x) 11270638

[bib18] Hirata T, Koga K, Johnson TA, et al. 2018 Japanese GWAS identifies variants for bust-size, dysmenorrhea, and menstrual fever that are eQTLs for relevant protein-coding or long non-coding RNAs. Sci Rep 8 8502. (10.1038/s41598-018-25065-9) 29855537 PMC5981393

[bib19] Hsu PS, Liu CH, Yang CJ, et al. 2024 Reward system neurodynamics during menstrual pain modulated by COMT Val158Met polymorphisms. Front Mol Neurosci 17 1457602. (10.3389/fnmol.2024.1457602) 39290829 PMC11405383

[bib20] Hu Z, Tang L, Chen L, et al. 2020 Prevalence and risk factors associated with primary dysmenorrhea among Chinese female university students: a cross-sectional study. J Pediatr Adolesc Gynecol 33 15–22. (10.1016/j.jpag.2019.09.004) 31539615

[bib21] Iacovides S, Avidon I & Baker FC 2015 What we know about primary dysmenorrhea today: a critical review. Hum Reprod Update 21 762–778. (10.1093/humupd/dmv039) 26346058

[bib22] Jones AV, Hockley JRF, Hyde C, et al. 2016 Genome-wide association analysis of pain severity in dysmenorrhea identifies association at chromosome 1p13.2, near the nerve growth factor locus. Pain 157 2571–2581. (10.1097/j.pain.0000000000000678) 27454463 PMC5436737

[bib23] Ju H, Jones M & Mishra G 2014 The prevalence and risk factors of dysmenorrhea. Epidemiol Rev 36 104–113. (10.1093/epirev/mxt009) 24284871

[bib24] Kajitani T, Maruyama T, Asada H, et al. 2013 Possible involvement of nerve growth factor in dysmenorrhea and dyspareunia associated with endometriosis. Endocr J 60 1155–1164. (10.1507/endocrj.ej13-0027) 23883529

[bib25] Kyathanahalli CN, Tu FF & Hellman KM 2025 Inflammatory mechanisms of dysmenorrhea: novel insights from menstrual effluent in an adolescent cohort. BJOG 132 1626–1634. (10.1111/1471-0528.18275) 40631483 PMC12258968

[bib26] Lee L-C, Tu C-H, Chen L-F, et al. 2014 Association of brain-derived neurotrophic factor gene Val66Met polymorphism with primary dysmenorrhea. PLoS One 9 e112766. (10.1371/journal.pone.0112766) 25383981 PMC4226574

[bib27] Lee C-C, Lee M-TG, Huang IH, et al. 2022 Genome-wide association study of primary dysmenorrhea in the Taiwan Biobank validates associations near the NGF and IL1 gene loci. J Hum Genet 67 449–458. (10.1038/s10038-022-01023-2) 35351958

[bib28] Lewin GR, Ritter AM & Mendell LM 1993 Nerve growth factor-induced hyperalgesia in the neonatal and adult rat. J Neurosci 13 2136–2148. (10.1523/jneurosci.13-05-02136.1993) 8478693 PMC6576576

[bib29] Li Z, Chen J, Wang Y, et al. 2017 Common variants in ZMIZ1 and near NGF confer risk for primary dysmenorrhoea. Nat Commun 8 14900. (10.1038/ncomms14900) 28447608 PMC5414039

[bib30] Li Y, Zhou S, Huang Y, et al. 2024 Phosphatidylcholine’s influence on dysmenorrhea: conclusive insights from mendelian randomization analysis. Front Genet 15 1404215. (10.3389/fgene.2024.1404215) 39376740 PMC11456477

[bib31] Little J, Higgins JP, Ioannidis JP, et al. 2009 Strengthening the reporting of genetic association studies (STREGA): an extension of the STROBE statement. Ann Intern Med 150 206–215. (10.7326/0003-4819-150-3-200902030-00011) 19189911

[bib32] Liu S, Wei Z, Carr DF, et al. 2024 Deciphering the genetic interplay between depression and dysmenorrhea: a mendelian randomization study. Brief Bioinform 26 bbae589. (10.1093/bib/bbae589) 39592111 PMC11596086

[bib33] Marjoribanks J, Ayeleke RO, Farquhar C, et al. 2015 Nonsteroidal anti-inflammatory drugs for dysmenorrhoea. Cochrane Database Syst Rev 2015 Cd001751. (10.1002/14651858.cd001751.pub3) 26224322 PMC6953236

[bib34] Muluneh AA, Nigussie TS, Gebreslasie KZ, et al. 2018 Prevalence and associated factors of dysmenorrhea among secondary and preparatory school students in Debremarkos town, North-West Ethiopia. BMC Womens Health 18 57. (10.1186/s12905-018-0552-x) 29699536 PMC5921558

[bib35] Nacar MC, Nursal AF, Kuruca N, et al. 2022 A circadian rhythm gene (PER3) VNTR variant as possible risk factor in cohort of Turkish females with primary dysmenorrhea. Nucleosides Nucleotides Nucleic Acids 41 900–909. (10.1080/15257770.2022.2085743) 35707903

[bib36] Nacar MC, Yigit S, Ozsoy AZ, et al. 2023 MEFV gene pathogenic variants: a risk factor for dysmenorrhea in the Turkish population. Nucleosides Nucleotides Nucleic Acids 43 1–12. (10.1080/15257770.2023.2293074) 38133485

[bib37] Nica AC & Dermitzakis ET 2013 Expression quantitative trait loci: present and future. Philos Trans R Soc Lond B Biol Sci 368 20120362. (10.1098/rstb.2012.0362) 23650636 PMC3682727

[bib38] Nickel JC, Mills IW, Crook TJ, et al. 2016 Tanezumab reduces pain in women with interstitial cystitis/bladder pain syndrome and patients with nonurological associated somatic syndromes. J Urol 195 942–948. (10.1016/j.juro.2015.10.178) 26576710

[bib39] Olasore H, Adebisi A, Oyedeji T, et al. 2022 COX-2 gene -1195G>A polymorphism (rs689466) is associated with dysmenorrhea among Nigerian women. Gene Rep 27 101592. (10.1016/j.genrep.2022.101592)

[bib40] Olasore H, Oyedeji T, Oluwole-Banjo A, et al. 2023 Glu298Asp polymorphism of the endothelial nitric oxide synthase (eNOS) gene (rs1799983) is associated with dysmenorrhea. Hum Gene 37 201184. (10.1016/j.humgen.2023.201184)

[bib41] Ouzzani M, Hammady H, Fedorowicz Z, et al. 2016 Rayyan-a web and mobile app for systematic reviews. Syst Rev 5 210. (10.1186/s13643-016-0384-4) 27919275 PMC5139140

[bib42] Ozerdogan N, Sayiner D, Ayranci U, et al. 2009 Prevalence and predictors of dysmenorrhea among students at a university in Turkey. Int J Gynaecol Obstet 107 39–43. (10.1016/j.ijgo.2009.05.010) 19539288

[bib43] Ozsoy AZ, Cakmak B, Nacar MC, et al. 2015 Mthfr and IL-4 gene polymorphisms are not associated with primary dysmenorrhea in young adults. Int J Hum Genet 15 73–79. (10.1080/09723757.2015.11886255)

[bib44] Ozsoy AZ, Karakus N, Yigit S, et al. 2016 The evaluation of IL6 and ESR1 gene polymorphisms in primary dysmenorrhea. Immunol Investig 45 75–86. (10.3109/08820139.2015.1113426) 26700208

[bib45] Page MJ, Mckenzie JE, Bossuyt PM, et al. 2021 The PRISMA 2020 statement: an updated guideline for reporting systematic reviews. BMJ 372 n71. (10.1136/bmj.n71) 33782057 PMC8005924

[bib46] Petty BG, Cornblath DR, Adornato BT, et al. 1994 The effect of systemically administered recombinant human nerve growth factor in healthy human subjects. Ann Neurol 36 244–246. (10.1002/ana.410360221) 8053664

[bib47] Pluchino N, Mamillapalli R, Wenger JM, et al. 2020 Estrogen receptor-α immunoreactivity predicts symptom severity and pain recurrence in deep endometriosis. Fertil Steril 113 1224–1231.e1. (10.1016/j.fertnstert.2020.01.036) 32416979

[bib48] Powell AM, Chan WY, Alvin P, et al. 1985 Menstrual-PGF2 alpha, PGE2 and TXA2 in normal and dysmenorrheic women and their temporal relationship to dysmenorrhea. Prostaglandins 29 273–290. (10.1016/0090-6980(85)90208-4) 3856904

[bib49] Rahmioglu N, Mortlock S, Ghiasi M, et al. 2023 The genetic basis of endometriosis and comorbidity with other pain and inflammatory conditions. Nat Genet 55 423–436. (10.1038/s41588-023-01323-z) 36914876 PMC10042257

[bib50] Reid-Mccann R, Poli-Neto OB, Stein K, et al. 2025 Longitudinal association between dysmenorrhoea in adolescence and chronic pain in adulthood: a UK population-based study. Lancet Child Adolesc Health 9 766–775. (10.1016/S2352-4642(25)00213-5) 40902609

[bib51] Silberg JL, Martin NG & Heath AC 1987 Genetic and environmental factors in primary dysmenorrhea and its relationship to anxiety, depression, and neuroticism. Behav Genet 17 363–383. (10.1007/bf01068137) 3675527

[bib52] Singgih EL, Van Der Voet M, Schimmel-Naber M, et al. 2021 Investigating cytosolic 5'-nucleotidase II family genes as candidates for neuropsychiatric disorders in drosophila (114/150 chr). Transl Psychiatry 11 55. (10.1038/s41398-020-01149-x) 33462198 PMC7813868

[bib53] Söderman L, Edlund M & Marions L 2019 Prevalence and impact of dysmenorrhea in Swedish adolescents. Acta Obstet Gynecol Scand 98 215–221. (10.1111/aogs.13480) 30312470

[bib54] Tavallaee M, Joffres MR, Corber SJ, et al. 2011 The prevalence of menstrual pain and associated risk factors among Iranian women. J Obstet Gynaecol Res 37 442–451. (10.1111/j.1447-0756.2010.01362.x) 21208343

[bib55] Wells GA, Shea B, O’connell D, et al. 2000 The Newcastle–Ottawa scale (NOS) for assessing the quality of nonrandomised studies in meta-analyses. (https://ohri.ca/en/who-we-are/core-facilities-and-platforms/ottawa-methods-centre/newcastle-ottawa-scale)

[bib56] Woo H-Y, Kim K-H & Lim S-W 2010 Estrogen receptor 1, glutathione S-transferase P1, glutathione S-transferase M1, and glutathione S-transferase T1 genes with dysmenorrhea in Korean female adolescents. Korean J Lab Med 30 76–83. (10.3343/kjlm.2010.30.1.76) 20197727

[bib57] Wu D, Wang X, Chen D, et al. 2000 Metabolic gene polymorphisms and risk of dysmenorrhea. Epidemiology 11 648–653. (10.1097/00001648-200011000-00006) 11055624

[bib58] Zhao S, Wu W, Kang R, et al. 2021 Significant increase in depression in women with primary dysmenorrhea: a systematic review and cumulative analysis. Front Psychiatry 12 686514. (10.3389/fpsyt.2021.686514) 34421672 PMC8374105

[bib59] Zhao D, Zeng LF, Liang GH, et al. 2022 Does anti-nerve growth factor monoclonal antibody treatment have the potential to replace nonsteroidal anti-inflammatory drugs and opioids in treating hip or knee osteoarthritis? A systematic review of randomized controlled trials. EFORT Open Rev 7 470–480. (10.1530/eor-21-0103) 35900204 PMC9297056

