# Supplementary material for: The genetics of primary dysmenorrhoea: a systematic review
Source: Reprod Fertil. 2026 Jul 30;7(3):RAF260004. doi: 10.1530/RAF-26-0004 (PMC13452037; doi:10.1530/RAF-26-0004)
Supplement: Supplementary file 1 [file RAF-26-0004_supplementary_materials.pdf]

Appendix

Supplementary Material 1 – Medline Search Strategy

1. exp menstruation disturbances/ or exp dysmenorrhea/
2. ("Primary dysmenorrh?ea" or "Painful menstruation" or "Menstrual pain" or "Period pain" or "Painful period" or "Period cramp$1" or "Dysmenorrh?ea pain" or "Menstruation disturbance$1" or "Menstrual disorder").ab,ti.
3. 1 or 2
4. exp genetics/ or exp genetics, population/ or exp genomics/ or exp human genetics/
5. exp disease susceptibility/ or exp genetic predisposition to disease/ or exp anticipation, genetic/
6. ("Genetic$1" or "Polymorphism$1" or "Single nucleotide polymorphism$1" or "SNP$1" or "Allele$1" or "Gene*4" or "Gene expression" or Variant$1 or "Gene* Association" or "Genetic varia*5" or "Gene variant$1" or "Mutation$1" or Genotype or "Genetic susceptibility" or "Genome Wide Association Study" or "Genome-Wide Association Study" or GWAS or "Twin Study" or "Inheritance" or "Genetic predisposition" or "Genetic susceptibility" or Heritability or Transmission or "Genetic influence" or Replication or "Candidate gene").ab,ti.
7. 4 or 5 or 6
8. 3 and 7
9. adenomyosis.ab,ti.
10. amenorrh?ea.ab,ti.
11. menorrhagia.ab,ti.
12. oligomenorrh?ea.ab,ti.
13. 9 or 10 or 11 or 12
14. (((menstruation disturbances or dysmenorrhea or ("Primary dysmenorrh?ea" or "Painful menstruation" or "Menstrual pain" or "Period pain" or "Painful period" or "Period cramp$1" or "Dysmenorrh?ea pain" or "Menstruation disturbance$1" or "Menstrual disorder")) and (genetics or genetics, population or genomics or human genetics or (disease susceptibility or genetic predisposition to disease or anticipation, genetic) or ("Genetic$1" or "Polymorphism$1" or "Single nucleotide polymorphism$1" or "SNP$1" or "Allele$1" or "Gene*4" or "Gene expression" or Variant$1 or "Gene* Association" or "Genetic varia*5" or "Gene variant$1" or "Mutation$1" or Genotype or "Genetic susceptibility" or "Genome Wide Association Study" or "Genome-Wide Association Study" or GWAS or "Twin Study" or "Inheritance" or "Genetic predisposition" or "Genetic susceptibility" or Heritability or Transmission or "Genetic influence" or Replication or "Candidate gene"))) not (adenomyosis or amenorrh?ea or menorrhagia or oligomenorrh?ea)).ab,ti.
15. limit 14 to English language
