# Supplementary material for: The genetics of primary dysmenorrhoea: a systematic review
Source: Reprod Fertil. 2026 Jul 30;7(3):RAF260004. doi: 10.1530/RAF-26-0004 (PMC13452037; doi:10.1530/RAF-26-0004)
Supplement: Supplementary file 2 [file RAF-26-0004_supplementary_tables.pdf]

Supplementary Table 1.1 – Quality Assessment of Included Studies by STREGA Guidelines

Study	Title & Abstract		Introduction			Methods																			
	1a	1b	2	3a	3b	4	5	6a	6b	7a	7b	8b	9a	9b	10	11	12a	12b	12c	12d	12e	12f	12g	12h	12i	12j
**Case–control**																										
Lee 2014	+	+	+	+	-	+	-	+	+	-	+	-	-	n/a	+	+	+	+	+	+	-	+	-	-	-	-
Ozsoy 2015	+	+	+	+	+	+	-	+	+	-	-	-	-	n/a	-	+	+	n/a	-	+	-	-	-	-	-	-
Dogru 2016	-	+	+	+	+	+	-	+	-	-	-	-	-	n/a	+	n/a	+	n/a	-	-	-	+	-	-	-	-
Ozsoy 2016	-	+	+	+	+	+	-	+	+	-	-	-	-	n/a	+	-	+	+	-	+	-	+	-	-	-	-
Esen 2020	+	+	+	+	-	-	-	+	-	+	-	-	-	n/a	+	+	+	n/a	-	-	-	-	-	-	-	-
Nacar 2022	-	+	+	+	-	+	-	+	+	-	-	-	-	n/a	+	+	+	n/a	-	-	-	-	-	-	-	-
Donayeva 2023	+	+	+	+	-	+	+	+	+	-	-	-	-	n/a	+	+	+	n/a	-	+	-	-	-	-	-	-
**Cross-sectional**																										
Wu 2000	-	+	+	+	-	+	-	+	n/a	-	-	-	-	n/a	+	+	+	n/a	-	n/a	-	-	-	-	-	-
Woo 2010	-	+	+	+	+	-	-	+	n/a	+	+	-	-	n/a	+	+	+	+	-	-	-	+	-	-	-	-
Olasore 2022	-	+	+	+	-	+	-	-	n/a	-	-	-	-	n/a	-	+	+	n/a	-	n/a	-	+	-	-	-	-
Olasore 2023	-	+	+	+	-	+	-	+	n/a	-	-	-	-	n/a	+	+	+	n/a	-	n/a	-	+	-	-	-	-
**GWAS**																										
Jones 2016	+	+	+	+	+	+	-	n/a	n/a	-	+	-	-	n/a	-	n/a	-	+	-	n/a	-	n/a	+	-	+	+
Li 2017	+	+	+	+	-	+	-	n/a	n/a	-	+	-	-	n/a	+	n/a	+	+	-	n/a	+	n/a	+	+	-	-
Hirata 2018	+	+	+	+	+	+	-	n/a	n/a	-	+	-	-	n/a	+	n/a	+	+	-	n/a	-	n/a	+	-	-	-
Lee 2022	+	+	+	+	-	+	-	n/a	n/a	-	+	-	-	n/a	+	n/a	+	+	-	n/a	-	n/a	+	-	-	-

<u>Supplementary Table 1.2 – Quality Assessment of Included Studies by STREGA Guidelines</u>

Study	Results														Discussion			Other info	
	13a	13b	13c	14a	14b	14c	15	16a	16b	16c	16d	17a	17b	17c	18	19	20	21	22
**Case–control**																			
Lee 2014	+	+	-	-	-	n/a	+	+	+	-	-	+	n/a	+	+	+	+	+	+
Ozsoy 2015	-	-	-	-	-	n/a	+	+	+	-	-	+	+	n/a	+	-	+	-	n/a
Dogru 2016	-	-	-	-	-	n/a	+	+	-	-	-	+	+	n/a	+	+	+	+	-
Ozsoy 2016	-	-	-	-	-	n/a	+	+	-	-	-	+	+	n/a	+	-	+	-	-
Esen 2020	-	-	-	+	-	n/a	+	+	+	-	-	n/a	n/a	n/a	+	+	+	-	n/a
Nacar 2022	-	-	-	-	-	n/a	+	+	n/a	-	-	+	n/a	n/a	+	+	+	+	n/a
Donayeva 2023	-	-	-	-	-	n/a	+	+	n/a	-	-	+	n/a	n/a	+	+	+	+	n/a
**Cross-sectional**																			
Wu 2000	+	+	-	-	-	n/a	+	+	+	-	+	+	+	n/a	+	+	+	+	+
Woo 2010	+	+	-	-	-	n/a	+	+	+	-	-	+	+	n/a	+	+	+	-	+
Olasore 2022	-	-	-	-	-	n/a	+	+	n/a	-	-	+	n/a	n/a	+	-	+	-	n/a
Olasore 2023	-	-	-	-	-	n/a	+	+	n/a	-	-	+	n/a	n/a	+	+	+	-	n/a
**GWAS**																			
Jones 2016	-	-	-	-	-	n/a	n/a	n/a	n/a	n/a	+	+	+	+	+	+	+	-	+
Li 2017	-	-	-	-	-	n/a	n/a	n/a	n/a	n/a	-	+	+	+	+	+	+	+	+
Hirata 2018	-	-	-	-	-	n/a	n/a	n/a	n/a	n/a	-	+	+	+	+	-	+	-	-
Lee 2022	+	+	+	-	-	n/a	n/a	n/a	n/a	n/a	-	+	+	+	+	+	+	+	+

Supplementary Table 2 – Quality Assessment of Included Case-Control Studies by Newcastle-Ottawa Scale (NOS)

**Study**	**SELECTION**				**COMPARABILITY**	**EXPOSURE**			**Total stars**	**Risk of bias**
	Is the case definition adequate?	Representativeness of the Cases	Selection of Controls	Definition of Controls	Comparability of Cases and Controls on the basis of Design or Analysis	Ascertainment of Exposure	Same Method of Ascertainment for Cases and Controls	Non-response Rate		
Lee 2014	*	-	-	*	-	*	*	-	4	Medium
Ozsoy 2015	-	-	-	*	-	-	*	-	2	High
Dogru 2016	*	*	-	-	-	*	-	-	3	High
Ozsoy 2016	*	*	-	-	-	-	*	-	3	High
Esen 2020	*	*	-	-	-	-	*	-	3	High
Nacar 2022	-	*	*	*	-	-	-	-	3	High
Donayeva 2023	*	*	*	*	-	*	-	-	5	Medium
